# Synovial Chondromatosis-Induced Monoarticular Knee Arthritis: Challenges With Arthroscopic Synovectomy on Late Presentation

**DOI:** 10.7759/cureus.30332

**Published:** 2022-10-15

**Authors:** Lavindra Tomar, Rakesh C Arya, Gaurav Govil, Pawan Dhawan

**Affiliations:** 1 Department of Orthopedics, Max Super Specialty Hospital, Delhi, IND; 2 Department of Orthopedics, Max Super Speciality Hospital, Delhi, IND

**Keywords:** arthritis and orthopaedic rheumatology, painful knee, arthroscopy synovectomy, monoarticular knee pain, synovial chondromatosis

## Abstract

Unilateral painful swelling of the knee is one of the most common orthopedic presentations. Monoarticular synovitis of the knee may be present due to inflammation, trauma, age-related degeneration, or tumor pathology. Primary synovial chondromatosis (PSC) is an uncommon cause.

A 40-year-old female presented with painful swelling of her left knee for around nine months. She had a flexion deformity of her left knee with minimal hydrops. The radiograph showed speckled calcifications and osteopenia with a reduction of medial joint space. MRI imaging confirmed synovitis with calcifications. She underwent an arthroscopic synovectomy for her management. Her biopsy was consistent with synovial chondromatosis. The initial progression was favorable to allowing independent, unsupported, pain-free activities of daily routine. At three months, however, there was disease progression, causing limitation of knee movements and the need for a walker for support.

An uncommon cause of synovitis presents at a late stage with a delay in the early diagnosis and early recognition. PSC is considered a benign lesion with good functional outcomes after arthroscopic surgery. Recurrence and poor functional outcome possibly suggest aggressive disease.

A delayed diagnosis and late presentation may be susceptible to recurrence and poor functional outcome, even after an adequate arthroscopic debridement and rigorous post-operative rehabilitation program.

## Introduction

The painful swelling of a single knee joint is one of the most common orthopedic outpatient presentations. The common causes of monoarticular synovitis may include trauma, infection, arthritis, tumors, or reactive affection [1]. However, the absence of any supportive diagnostic or imaging findings may pose a diagnostic challenge for rare affections.

Primary synovial chondromatosis (PSC) affecting the knee characteristically has cartilaginous nodules, usually intraarticular, forming loose bodies of unknown etiology [2-4]. A benign disorder that may cause synovial metaplasia with monoarticular knee synovitis [5]. Such an uncommon etiology poses diagnostic and management dilemmas. The literature review presented a few case reports of PSC affecting the knee joint as rare presentations [3,5-7]. Arthroscopic synovectomy has given good functional outcomes. Any delay in diagnosis presents numerous challenges in management with eventual poor outcomes and functional joint impairment.

We present a case report highlighting the challenges with arthroscopic management of monoarticular knee synovitis presenting late with flexion deformity. Additionally, a review of the relevant literature on the diagnosis and management options for a monoarticular PSC of the knee has been presented. Recurrence with disease progression may cause arthritis with poor joint function.

## Case presentation

A 40-year-old woman from a rural background had a history of an insidious onset of pain in her left knee with difficulty in walking, standing, and doing her routine activities for around nine months. A local medical center in her native village diagnosed her with left knee synovitis. A workup with radiography of the knee revealed no abnormality. Painkillers and analgesics were advised along with calcium and vitamin D supplements for her medical management. Non-ambulatory status was maintained for an initial period of six weeks. Synovitis knee was diagnosed erroneously as reactive synovitis and conservative measures were initiated. There was no relief in pain and the left knee progressed to develop a flexion deformity. She was unable to ambulate without support, and any attempted movements of her left knee were resisted. In the last three months, her ambulatory status deteriorated further due to persistent progressive left knee pain and deformity, making her unable to walk for any daily routine activities without pain. Recent radiographs at the time of presentation revealed speckled calcification along the knee with a marked reduction in joint space with the knee in a flexion attitude. She presented us with the relevant concern for treatment.

There was severe pain in her left knee and marked difficulty in doing her activities of daily living, including using the restroom without support for the last two months. There was no past history of trauma, fever, or pulmonary tuberculosis. There was a wasting of thigh muscles, which prevented her from ambulating without support. Her body mass index was low. There was generalized tenderness over the left knee and minimal hydrops with a fixed flexion deformity of the left knee with a 10 to 20-degree range of motion. Any further attempted movement of the limb was dissuaded and resisted. The right knee was normal on examination. The patient was able to do straight leg raises on the right side but not on the left side. The hip rotations were within normal ranges. There was no distal neurovascular deficit in either of the lower limbs.

**Figure 1:**
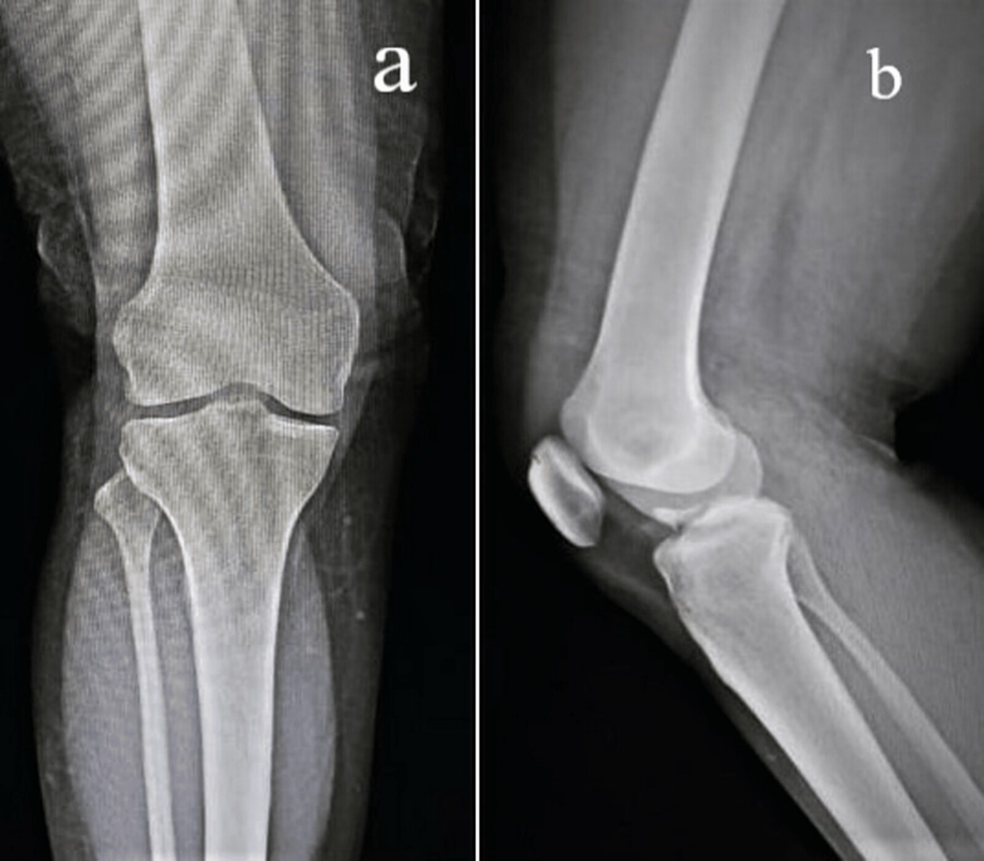
Radiograph of the left knee in AP (a) and lateral (b) views with maintained joint space, and articular cartilage.

Sequential radiographs show disease progression. The initial radiograph showed normal features with maintained joint space and articular margins (Figure 1).

**Figure 2:**
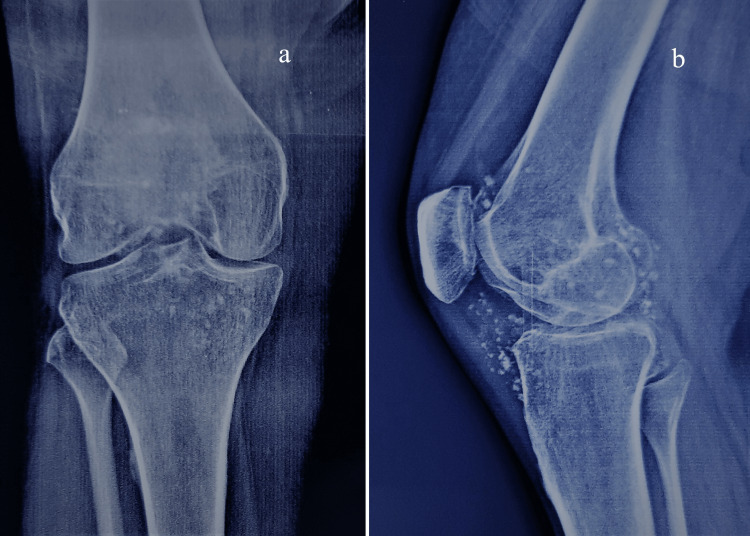
Radiograph of left knee in AP (a) and lateral (b) view after three months with calcification and preserved joint space.

It was consistent with Milgram's "early stage" where active intra-synovial disease presents without loose bodies [8]. The second follow-up radiograph at three months of left knee pain showed calcification in the joint area with no significant reduction in joint space, which correlated to Milgram's "transitional phase" with active disease and loose bodies (Figure 2).

**Figure 3:**
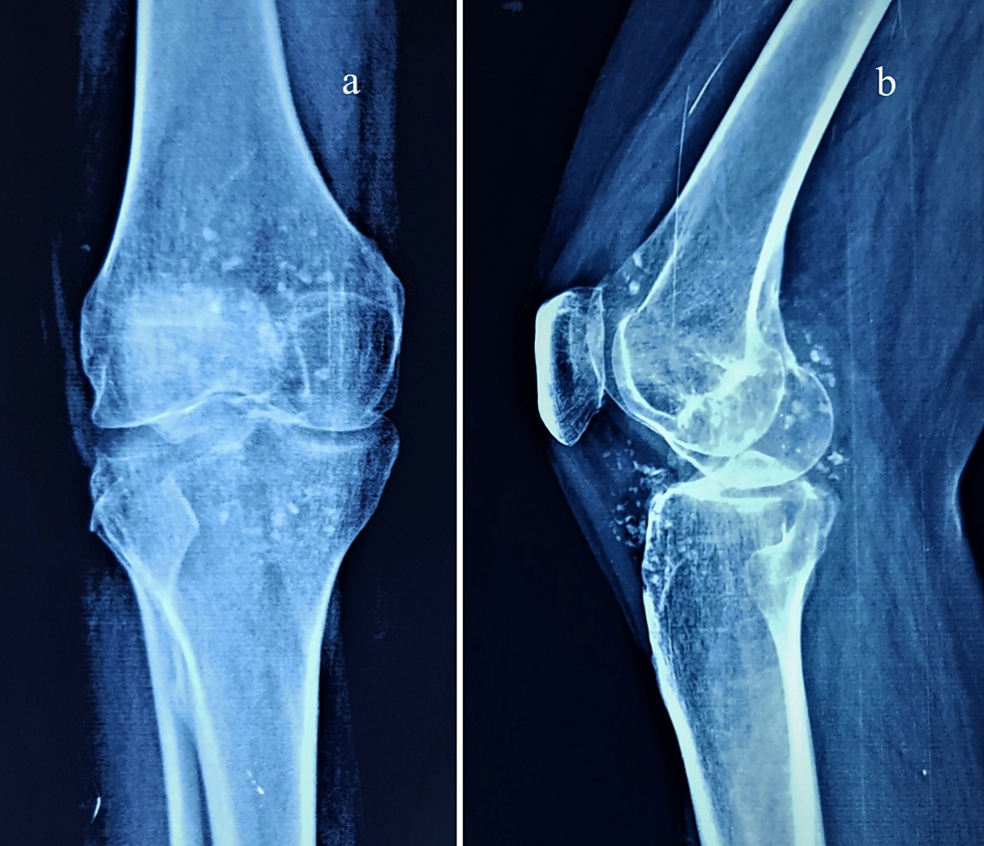
Radiograph of left knee in AP (a) and lateral (b) view after nine months of disease onset with speckled calcification, diminished joint space, and flexion deformity.

The follow-up radiograph at nine months showed speckled calcification around the knee joint, likely both intra-articular and extra-articular, with decreased joint space. The knee arthritis had progressed to Milgram's "late stage" (Figure 3).

**Figure 4:**
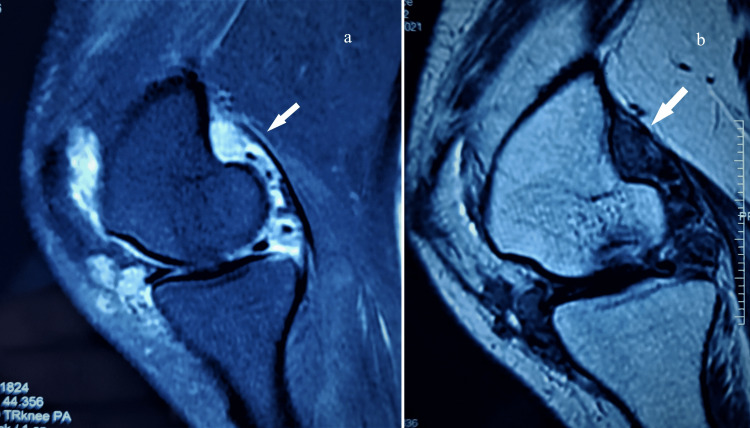
MRI of left knee shows diffuse nodular synovial hypertrophy in T2W (a) and TIW (b) images along tibiofemoral joint with multiple T2W hypointense foci showing blooming on GRE images within the knee joint (white arrow).

Magnetic resonance imaging (MRI) of the left knee shows diffuse nodular synovial hypertrophy along the tibiofemoral joint, with multiple T2W hypointense foci showing blooming on GRE images within the knee joint (Figure 4).

**Figure 5:**
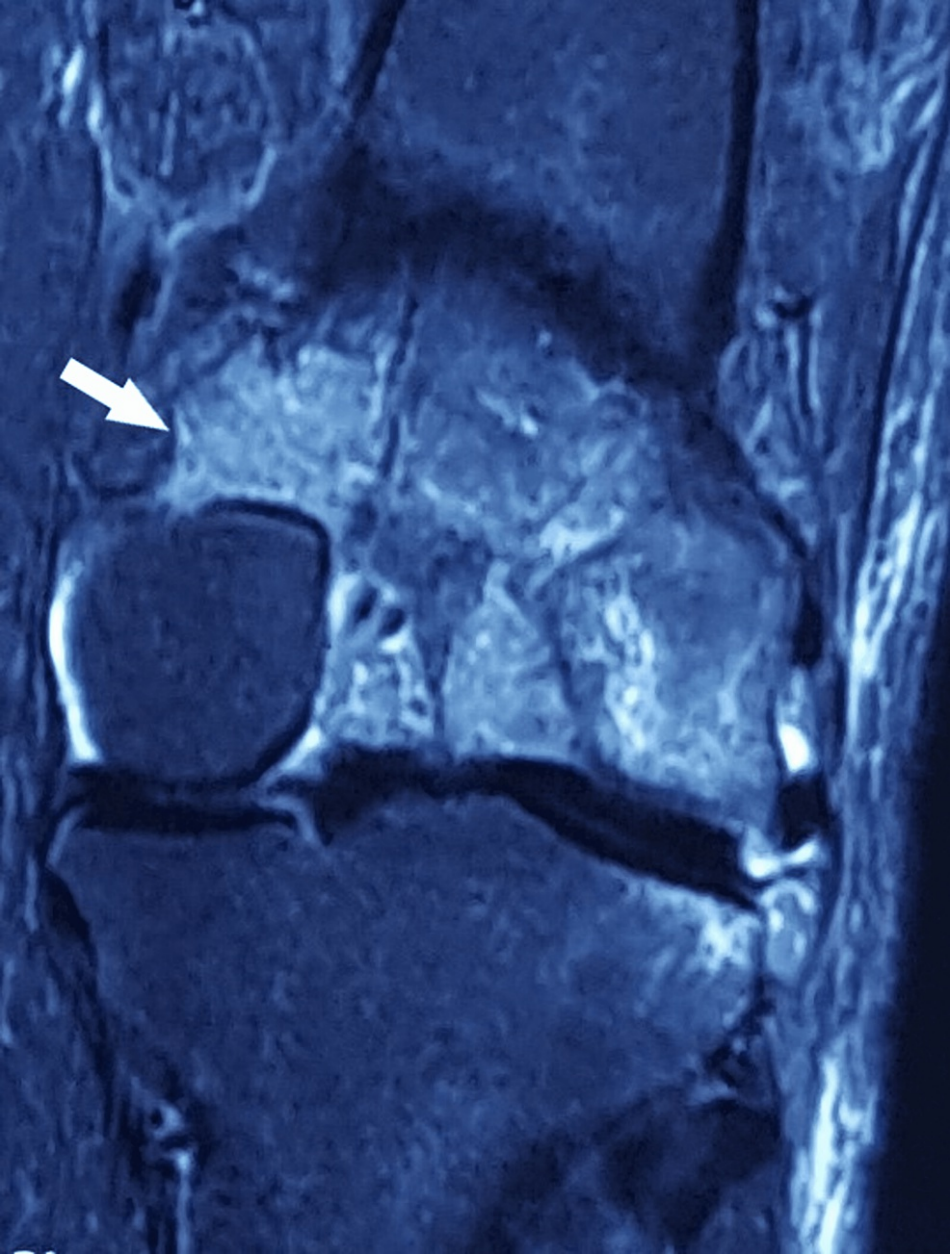
MRI of left knee shows marrow oedema involving lateral tibial condyle and posterior aspect of lateral femoral condyle (marked with white arrow).

MRI also presents with marrow oedema involving the lateral tibial condyle and the posterior aspect of the lateral femoral condyle (Figure 5).

The erythrocyte sedimentation rate was measured at 35 mm/hour and C-reactive protein was qualitatively negative. Her pre-operative investigations included normal calcium, phosphorus, alkaline phosphatase, parathyroid hormone, rheumatoid factor, and serum 25-hydroxyvitamin D levels. Informed consent has been obtained from the patient to publish the data.

**Figure 6:**
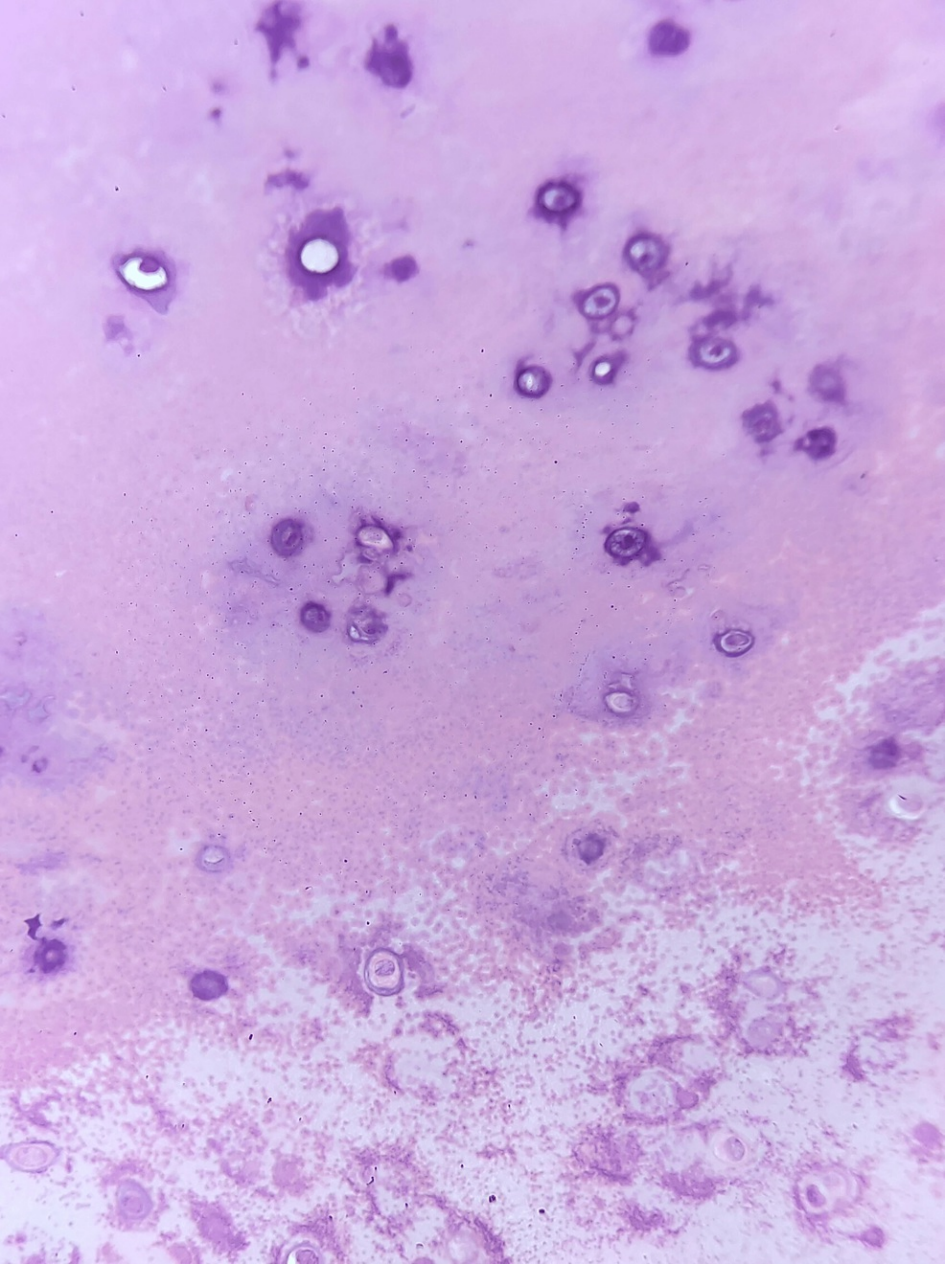
Histopathology microscopic examination consistent with synovial chondromatosis.

The options of open and arthroscopic surgery were discussed with the patient and she was subsequently planned for an arthroscopic surgery. The arthroscopic surgery allowed adequate clearance of the diseased synovial tissue. The goals were the removal of nodular deposits, synovial debulking, and tissue biopsy. The option of manipulation of the knee was suggested after the assessment of the flexion deformity under anesthesia. It was a single-stage procedure done using spinal anesthesia. Manipulation for passive correction of flexion was undertaken with subsequent left knee diagnostic arthroscopy. A standard lateral parapatellar portal was established; however, due to the flexion deformity, it was a challenge to negotiate the scope into the suprapatellar region. Once in the suprapatellar pouch, another standard medial portal was used to place an oscillating shaver blade to remove the suprapatellar adhesions. There were multiple nodules with gritty consistency, synovial hyperemia, and hypertrophy. The joint was carefully debrided to prevent abnormal bleeding. On subsequent exploration, the joint revealed multiple impregnated synovial nodules of different sizes with a thick gritty consistency, which were difficult to remove. The medial and lateral gutters were cleared of villous projections with the debridement of nodular deposits. There were intra-articular adhesions, likely fibrosis, consistent with non-usage of the joint. The arthroscopic evaluation of the meniscus and cruciate ligaments was normal. The joint cartilage was fairly preserved with no evidence of advanced cartilage degeneration. Removal of multiple nodules, subtotal synovectomy, and debridement of the adjacent fibrous tissue were done with caution. However, all the adherent loose bodies could not be removed. A per-operative extension of the knee to within 5 degrees of extensor lag with flexion of up to 100 degrees was achieved. A long knee immobilizer was applied post-operatively to maintain the extension of the knee. The total duration of surgery was 62 minutes. The patient was ambulated with walker support from the second post-operative day. Post-operatively, she was given therapeutic doses of vitamin D and calcium supplements. She was discharged on the third day of the surgery. There were no immediate post-operative complications. Histopathological examination of loose bodies showed synovial tissue with dispersed chondrocytes in lacunae and occasional binucleate chondrocytes over a chondroid matrix, suggestive of PSC (Figure 6).

Mobilization and knee bending exercises were initiated in the immediate post-operative period. Subsequently, after an initial one-week, a continuous passive mobilization machine was applied and a flexion range of about 80 degrees was achieved within the next two weeks. The quadriceps strengthening was done with muscle stimulation, persistent passive mobilization, and active leg raises under a vigorous rehabilitation program to increase her range of movements up to 100 degrees of flexion.

**Figure 7:**
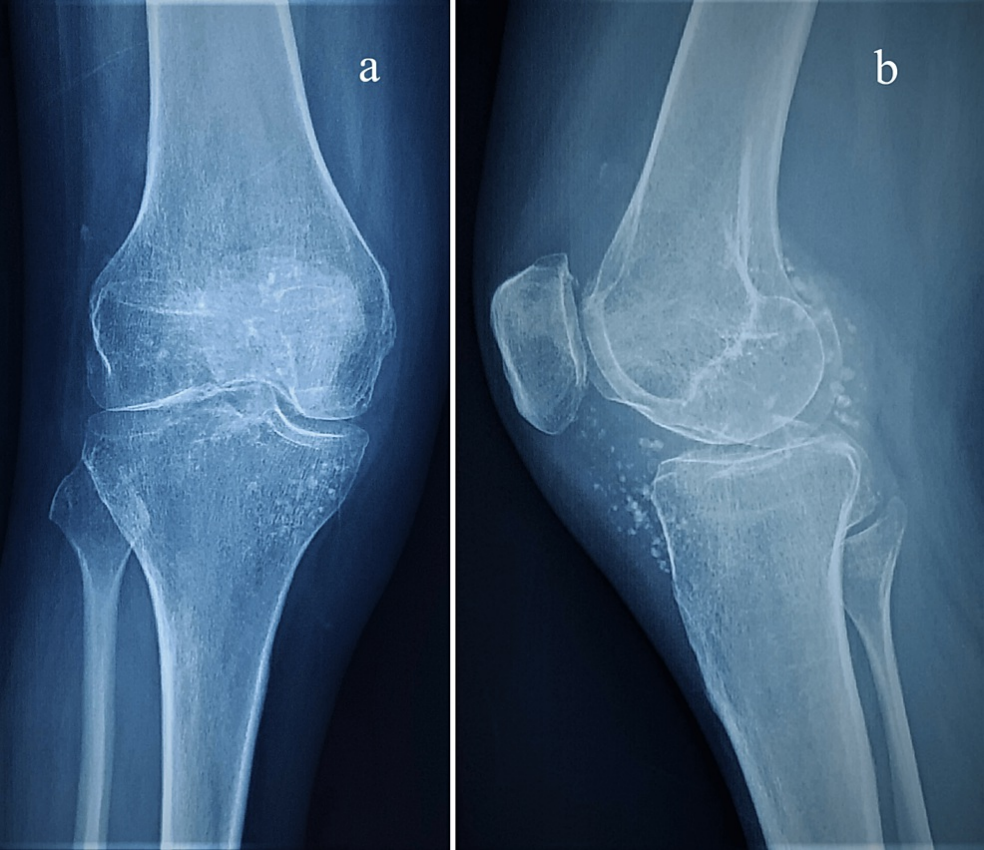
Radiograph of left knee in AP (a) and lateral (b) view after three months of surgery shows few calcifications more in the extraarticular region with flexion deformity.

At the three-month follow-up, however, the range of knee motion diminished, and a recurrence of pain was noted. She was gradually unable to mobilize without support, though she had no limb length discrepancy or gait abnormality. Radiologically, there were extraarticular calcifications with flexion deformity (Figure 7).

**Figure 8:**
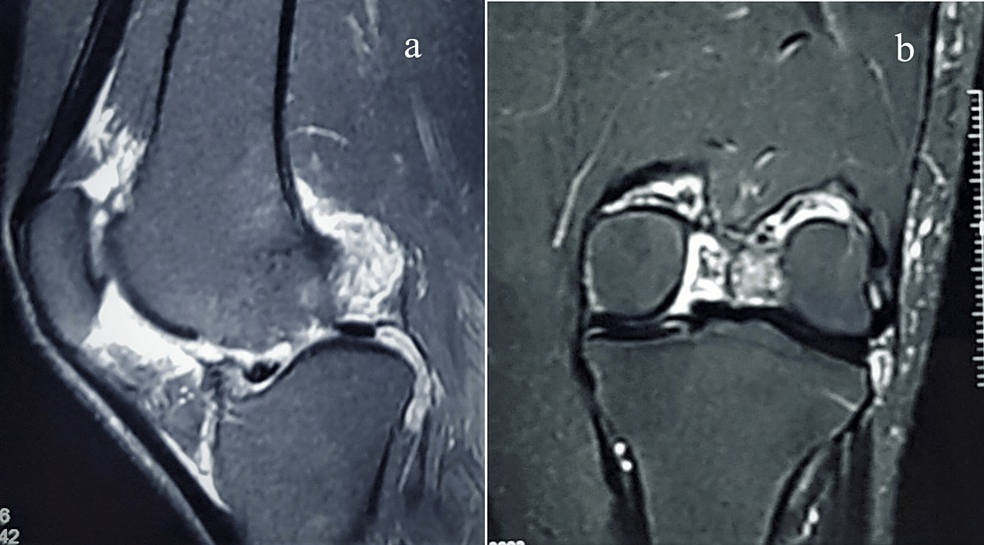
MRI of left knee in sagittal (a) and coronal (b) sections shows calcifications with deposits in synovium.

A repeat MRI was done and there were recurrent calcific deposits in the synovium with cartilage degradation (Figure 8).

At the six-month follow-up, an open synovial debridement and redo biopsy were advised to also rule out malignant transformation if any. An option of radiotherapy was also discussed. However, any further intervention was resisted, and she continued to walk with support for her daily routine activities. Subsequently, she was lost to follow-up.

## Discussion

The knee joint affection in PSC presents predominantly with intra-articular affection, though extra-articular or combined affection may also be present rarely [2]. PSC has been recognized as 'snowstorm knee', 'Reichel syndrome', and literature presents with only a few case reports [7,9]. Males are predominantly affected more as compared to females and commonly there is affection in the age group of 20 to 50 years with affection in children being rarely reported [2-7,10].

The diagnosis requires a thorough history taking and examination to exclude the common causes of affection for monoarticular knee synovitis. Radiology can be more expressive in its initial presentation as subtle calcifications in and around the knee joint, either intra-articular or extra-articular, can be seen [2]. Intraarticular lesions form in 70 to 95% of cases, and extraarticular loose bodies form in 20% to 50% of affections [7]. However, lack of calcification in nodules during the early stages of presentation may cause 30% of the cases likely to be missed in the initial stages [4,10]. Certain patterns of calcification signifying chondroid origin have been described as 'dot‑and‑comma', 'ring‑and‑arc', or 'popcorn‑like' calcification [2,7,11]. Later, there are arthritic changes with the progression of the cartilage destruction with joint space obliteration, further immobilizing the joint [2]. The case in the study highlights the disease progression as evident on the sequential radiographs.

MRI of the knee joint presents with hypointense calcification and signal alterations from the low signal intensity on T1W to the high signal intensity on the T2W sequences [2,3,12]. MRI findings are based on the mineralization status to present various calcifications [7]. The differential diagnosis includes rheumatoid arthritis, psoriatic arthritis, inflammatory arthritis, tumoral calcinosis, villous nodular synovitis, synovial chondrosarcoma, and lipoma arborescence [9,10]. MRI with additional use of contrast can differentiate the pathologies largely based on the changes in the synovium, calcification patterns, mineralization, and bony erosions [9-11]. The MRI in our case at the time of presentation suggested Milgram’s intermediary stage of disease with intraarticular and extraarticular lesions with preserved cartilage.

The treatment for PSC has no medical management. Surgical excision, either open or arthroscopic synovectomy, provides relief [2,9]. Arthroscopic removal of loose bodies alone or in conjunction with synovectomy has given similar outcomes [13]. The recurrence rate has been reported as 3-23% [5,6]. Radiotherapy can be considered rarely for recurrent or resistant lesions [4,5]. Though malignant transformation to synovial chondrosarcoma has been reported rarely, a cautious approach is needed to identify it [11]. The recurrences or resistant cases are suspicious cases for transformation, and the differentiation is largely based on subjective assessment of the degree of cellularity, nature of the matrix, and chondrocyte features [10,11]. We had a recurrence in our case, though no radiological evidence of malignant transformation on a repeat MRI could be confirmed. The indication was for an active disease process affecting the functional outcome.

## Conclusions

An early presentation of synovial chondromatosis generally responds favorably to arthroscopic management. However, a late presentation with a delayed diagnosis may present a poor outcome even after an adequate arthroscopic synovectomy. The recurrence may necessitate revision arthroscopic surgery or consideration of knee arthroplasty.

An initial joint-saving surgical intervention should be offered first. The treatment should be planned further based on the clinical presentation and the stage of the disease activity.
